# Na_1.85_Mg_1.85_In_1.15_(PO_4_)_3_ and Ag_1.69_Mg_1.69_In_1.31_(PO_4_)_3_ with alluaudite-type structures

**DOI:** 10.1107/S2056989018011799

**Published:** 2018-08-24

**Authors:** Ahmed Ould Saleck, Abderrazzak Assani, Mohamed Saadi, Cyrille Mercier, Claudine Follet, Lahcen El Ammari

**Affiliations:** a Laboratoire de Chimie Appliquée des Matériaux, Centre Sciences des Matériaux, Faculty of Sciences, Mohammed V University in Rabat, Avenue Ibn Batouta, BP 1014, Rabat, Morocco; b Université de Valenciennes, EA 2443 – LMCPA – Laboratoire des Matériaux Céramiques et Procédés Associés, F-59313 Valenciennes, France

**Keywords:** crystal structure, mixed-metal phosphate, solid-state reaction, disorder, alluaudite

## Abstract

Single crystals of two new phosphates, sodium magnesium indium(III) tris­(orthophosphate) and silver magnesium indium(III) tris­(orthophosphate), were obtained from solid-state reactions. The two phosphates are isotypic and exhibit alluaudite-type structures. They are characterized by a cationic disorder of the Mg and In sites and a partial occupation of the Na and Ag sites, respectively. The structure of both phosphates is made up of chains of edge-sharing [(Mg,In)O_6_] octa­hedra extending parallel to [10]. Adjacent chains are linked by PO_4_ tetra­hedra to form a three-dimensional framework delimiting two types of channels parallel to [001] in which the monovalent cations are situated. The coordination numbers of the Na^+^ cations are 6 and 8, and for both Ag^+^ cations 6. The corresponding coordination spheres are considerably distorted.

## Abstract 2

The two orthophosphates, Na_1.85_Mg_1.85_In_1.15_(PO_4_)_3_ and Ag_1.69_Mg_1.69_In_1.31_(PO_4_)_3_ adopt alluaudite-type structures. Edge-sharing [(In,Mg)O_6_] octa­hedra are linked together by PO_4_ tetra­hedra, leaving channels in which the Na^+^ or Ag^+^ cations are situated..

## Chemical context   

The crystal structure of the mineral alluaudite was determined by Moore (1971 ▸). Since then, many new members of this structure type, including phosphates, arsenates, molybdates, sulfates and, more recently, vanadates have been synthesized and structurally characterized. The growing inter­est in these kinds of materials is related to their inter­esting physical properties, in particular in electrochemistry and battery research. For example, the phosphate Na_2_Ni_2_Fe(PO_4_)_3_ (Essehli *et al.*, 2015 ▸) is a promising cathode in sodium batteries since its electrochemical behaviour is comparable to that of LiFePO_4_. In this context, alluaudite-type phosphates such as Na_1.67_Zn_1.67_Fe_1.33_(PO_4_)_3_ (Khmiyas *et al.*, 2015 ▸), Ag_1.655_Co_1.647_Fe_1.352_(PO_4_)_3_ (Bouraima *et al.*, 2017 ▸) and the vanadate (Na_0.7_)(Na_0.70_, Mn_0.30_) (Fe^3+^, Fe^2+^)_2_Fe^2+^(VO_4_)_3_ (Benhsina *et al.*, 2016 ▸) have been investigated by our group.

In the present work, the synthesis and structure determination of two new magnesium-based alluaudite-type phosphates with composition Na_1.85_Mg_1.85_In_1.15_(PO_4_)_3_ (I) and Ag_1.69_Mg_1.69_In_1.31_(PO_4_)_3_ (II) are reported.

## Structural commentary   

**Figure 1:**
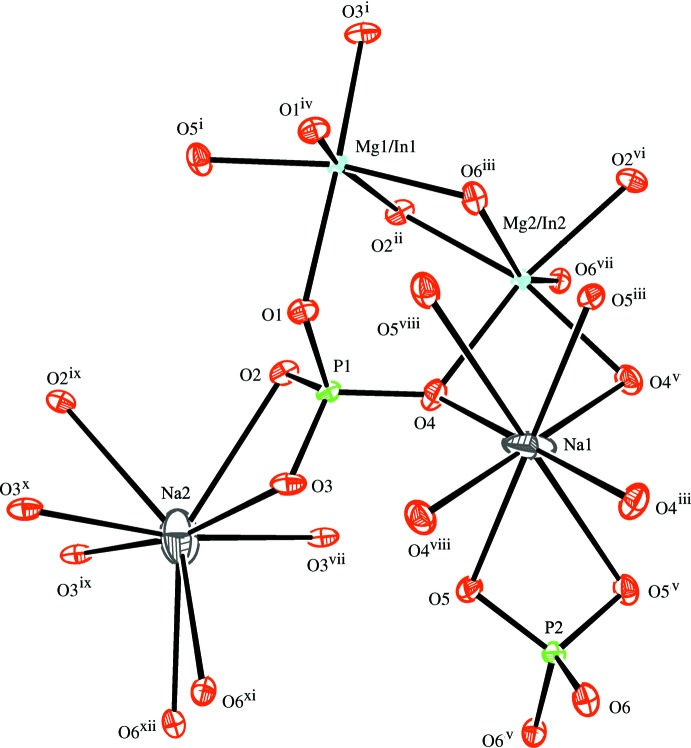
The principal building units in the structure of Na_1.85_Mg_1.85_In_1.15_(PO_4_)_3_, (I). Displacement ellipsoids are drawn at the 50% probability level. [Symmetry codes: (i) −*x* + 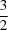, *y* + 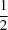, −*z* + 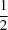; (ii) −*x* + 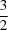, −*y* + 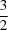, −*z* + 1; (iii) −*x* + 1, −*y* + 1, −*z*; (iv) −*x* + 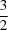, −*y* + 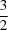, −*z*; (v) −*x* + 1, *y*, −*z* + 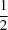; (vi) *x* − 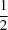, −*y* + 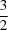, *z* − 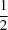; (vii) *x*, −*y* + 1, *z* + 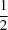; (viii) *x*, −*y* + 1, *z* − 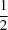; (ix) −*x* + 2, *y*, −*z* + 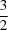; (*x*) −*x* + 2, −*y* + 1, −*z* + 1; (xi) *x* + 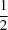, −*y* + 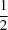, *z* + 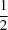; (xii) −*x* + 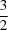, −*y* + 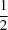, −*z* + 1.]

**Figure 3:**
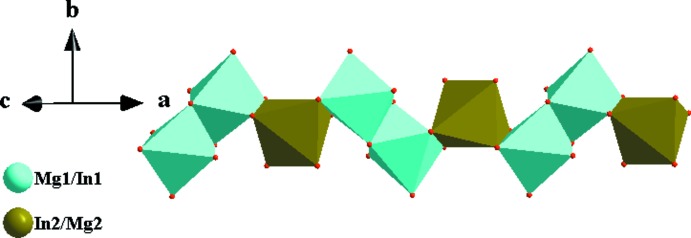
Edge-sharing [(Mg/In)2O_6_] octa­hedra and [(Mg/In)1)_2_O_10_] dimers forming an infinite chain extending parallel to [00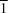]. Data taken from (I).

**Figure 4:**
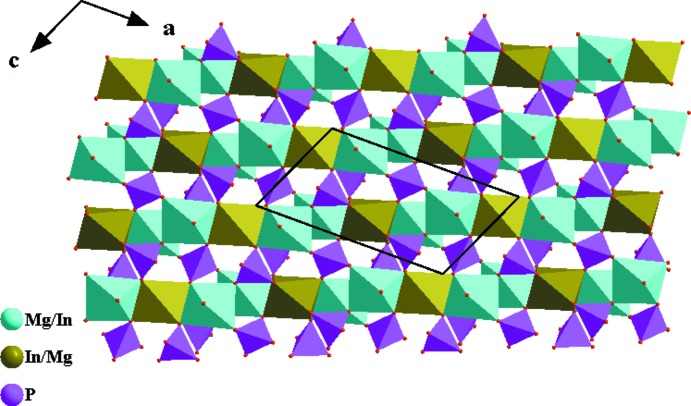
[(Mg/In)O_6_] octa­hedra and PO_4_ tetra­hedra forming a sheet extending parallel to (010). Data taken from (I).

**Figure 5:**
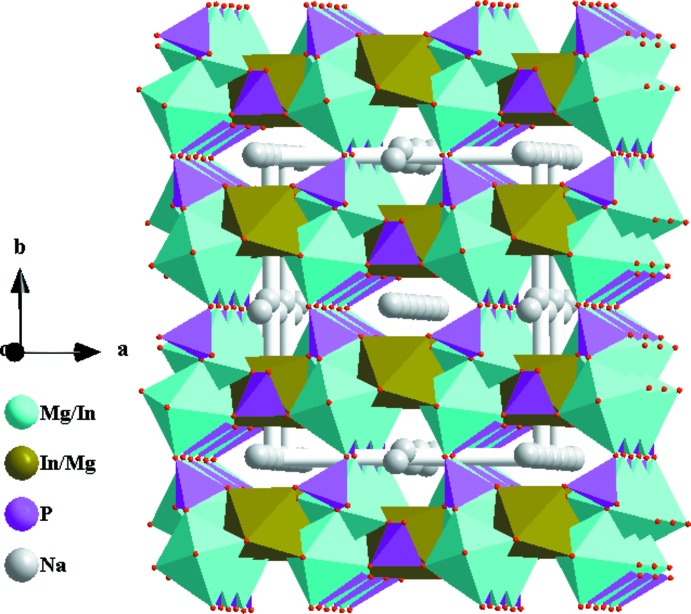
Polyhedral representation of the crystal structure of (I) showing Na^+^ cations situated in the two types of channels parallel to [001].

In the crystal structures of the two isotypic phosphates (I) and (II), site Na1 (Ag1) shows full occupancy and is located on an inversion centre (Wyckoff position 4*b*), and one mixed-occupied (Mg/In)2 site [occupancy ratio Mg:In = 0.51:0.49 for (I) and 0.314:0.686 for (II)], the second partially occupied Na2 (Ag2) site [occupancy 0.848 (9) for (I) and 0.6988 for (II)] and the P1 site are located on twofold rotation axes (4*e*) of space group type *C*2/*c*. There is another mixed-occupancy (Mg,In)1 site in a general position (8*f*) with occupancy ratios Mg:In = 0.68:0.32 for (I) and 0.687 (2):0.314 (2) for (II). This kind of cationic disorder is a characteristic feature of alluaudite-type structures. The principal building units in the crystal structures of (I) and (II) are [(Mg/In)1O_6_] and [(Mg,In)2O_6_] octa­hedra and two PO_4_ tetra­hedra (Figs. 1 ▸ and 2 ▸). Two [(Mg/In)1O_6_] octa­hedra are linked together by a common edge into an [(Mg/In)1)_2_O_10_] dimer. These dimers are connected through edge-sharing with [(Mg/In)2O_6_] octa­hedra into undulating chains extending parallel to [10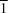] (Fig. 3 ▸). Adjacent chains are linked together by P1O_4_ and P2O_4_ tetra­hedra into (010) sheets, as shown in Fig. 4 ▸. Neighbouring sheets are finally fused into a three-dimensional framework structure by P1O_4_ tetra­hedra. This framework delimits two types of hexa­gonal channels oriented parallel to [001], in which the Na^+^ (for (I) or Ag^+^ (for (II) cations are located (Fig. 5 ▸). The Na—O distances fall in the range 2.307 (2)–2.960 (2) Å with coordination numbers of six for Na1 and eight for Na2, while those for Ag—O vary between 2.345 (2) and 2.963 (2) Å, with coordination numbers of six for both Ag^+^ cations.

## Database Survey   

The presence of disordered alkali metal or other cations in the channels of alluaudite-type structures is a concomitant feature of the cationic disorder at the octa­hedral sites, as observed for example in Cu_1.35_Fe_3_(PO_4_)_3_ (Warner *et al.*, 1993 ▸), (Na_0.38_,Ca_0.31_)MgFe_2_(PO_4_)_3_ (Zid *et al.*, 2005 ▸), K_0.53_Mn_2.37_Fe_1.24_(PO_4_)_3_ (Hidouri & Ben Amara, 2011 ▸), NaFe_3.67_(PO_4_)_3_ (Korzenski *et al.*, 1998 ▸), Na_1.25_Mg_1.10_Fe_1.90_(PO_4_)_3_ (Hidouri *et al.*, 2008 ▸), Na_1.50_Mn_2.48_Al_0.85_(PO_4_)_3_ (Hatert, 2006 ▸), Na_1.79_Mg_1.79_Fe_1.21_(PO_4_)_3_ (Hidouri *et al.*, 2003 ▸), Na_1.67_Zn_1.67_Fe_1.33_(PO_4_)_3_ (Khmiyas *et al.*, 2015 ▸) or Ag_1.655_Co_1.647_Fe_1.352_(PO_4_)_3_ (Bouraima *et al.*, 2017 ▸).

## Synthesis and crystallization   

Single crystals of (I) and (II) were grown by solid-state reactions. The starting mixtures comprising of Mg(NO_3_)_2_·6H_2_O (Sigma–Aldrich, 97%), InI_3_ (Ventron, 99%), NH_4_H_2_PO_4_ (Alfa Aesar, 98%), *A*NO_3_ (*A* = Na or Ag) (NaNO_3_: Acros Organics, 99%; AgNO_3_: Sigma–Aldrich, 99%) were weighted in molar ratios *A*:Zn:In:P = 2:2:1:3 and placed in a platinum cruicible. After inter­mediate grinding and temperature treatments at 573, 673, 773 and 873 K in a platinum crucible, both mixtures were heated at 1373 K above the melting temperatures. The cruicibles were then cooled slowly to 1093 K at a rate of 5 K h^−1^, followed by cooling to room temperature after switching off the furnace. Transparent, colourless crystals with a blocky form were isolated from the two final products. The bulk products were not checked for phase purity.

## Refinement   

Crystal data, data collection and structure refinement details are summarized in Table 1 ▸. In the initial stages of the refinements the occupancies of the disordered sodium (Na2) or silver (Ag2) sites were refined freely and the mixed-occupancy (Mg/In) sites were refined under consideration of full occupancy for each of these sites. The obtained occupancy rates of Mg:In were rounded and subsequently fixed for charge-neutrality of the compounds. The maximum and minimum electron densities are located 0.55 Å from Mg2 and 0.38 Å from P1 for (I) and 0.78 and 0.59 Å, respectively, from Ag2 for (II).

## Supplementary Material

Crystal structure: contains datablock(s) I, II, global. DOI: 10.1107/S2056989018011799/wm5457sup1.cif

Structure factors: contains datablock(s) I. DOI: 10.1107/S2056989018011799/wm5457Isup2.hkl

Structure factors: contains datablock(s) II. DOI: 10.1107/S2056989018011799/wm5457IIsup3.hkl

CCDC references: 1862981, 1862980

Additional supporting information:  crystallographic information; 3D view; checkCIF report

## Figures and Tables

**Figure 2:**
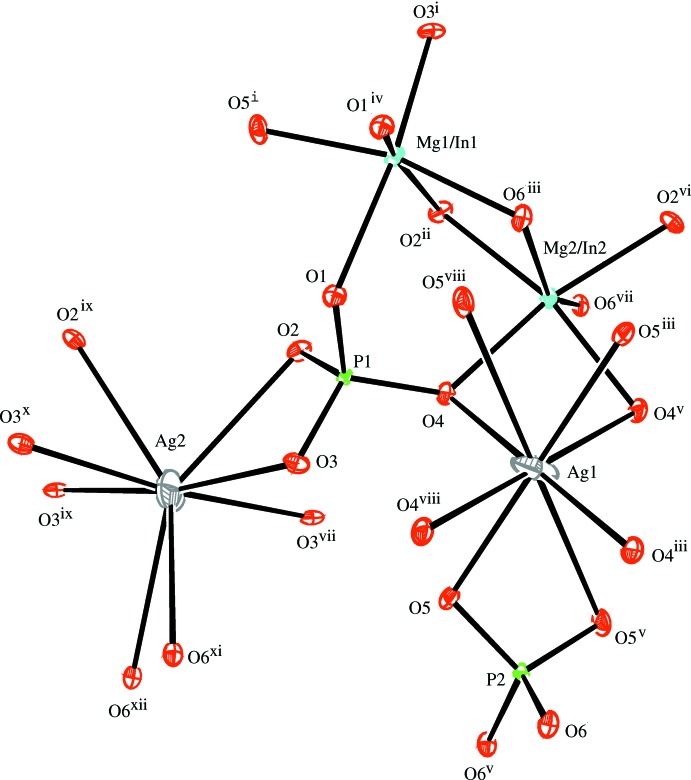
The principal building units in the structure of Ag_1.69_Mg_1.69_In_1.31_(PO_4_)_3_, (II). Displacement ellipsoids are drawn at the 50% probability level. Symmetry codes are as in Fig. 1 ▸.

**Table 1:** Experimental details

	(I)	(II)
Crystal data		
Chemical formula	Na_1.85_Mg_1.85_In_1.15_(PO_4_)_3_	Ag_1.69_Mg_1.69_In_1.31_(PO_4_)_3_
*M*_r_	504.46	658.40
Crystal system, space group	Monoclinic, *C*2/*c*	Monoclinic, *C*2/*c*
Temperature (K)	296	296
*a*, *b*, *c* (Å)	11.9796 (13), 12.6935 (13), 6.5239 (7)	12.0273 (3), 12.8120 (3), 6.5061 (2)
β (°)	114.555 (3)	114.519 (1)
*V* (Å^3^)	902.33 (17)	912.14 (4)
*Z*	4	4
Radiation type	Mo *K*α	Mo *K*α
μ (mm^−1^)	3.82	7.59
Crystal size (mm)	0.31 × 0.24 × 0.20	0.30 × 0.27 × 0.23

Data collection		
Diffractometer	Bruker X8 APEXII	Bruker X8 APEXII
Absorption correction	Multi-scan (*SADABS*; Krause *et al.*, 2015 ▸)	Multi-scan (*SADABS*; Krause *et al.*, 2015 ▸)
*T*_min_, *T*_max_	0.596, 0.748	0.404, 0.748
No. of measured, independent and observed [*I* > 2σ(*I*)] reflections	21364, 2076, 2012	13615, 1827, 1818
*R*_int_	0.026	0.027
(sin θ/λ)_max_ (Å^−1^)	0.819	0.781

Refinement		
*R*[*F* ^2^ > 2σ(*F* ^2^)], *wR*(*F* ^2^), *S*	0.024, 0.058, 1.29	0.022, 0.053, 1.25
No. of reflections	2076	1827
No. of parameters	97	97
Δρ_max_, Δρ_min_ (e Å^−3^)	0.64, −0.88	2.32, −1.36

Computer programs: *APEX2* and *SAINT* (Bruker, 2014 ▸), *SHELXS2016* (Sheldrick, 2015*a* ▸), *SHELXL2016* (Sheldrick, 2015*b* ▸), *ORTEP-3 for Windows* (Farrugia, 2012 ▸), *DIAMOND* (Brandenburg, 2006 ▸) and *publCIF* (Westrip, 2010 ▸).

## supplementary crystallographic information

### Sodium magnesium indium(III) tris(orthophosphate) (I) Crystal data

**Table:**

Na_1.85_Mg_1.85_In_1.15_(PO_4_)_3_	*F*(000) = 960
*M**_r_* = 504.46	*D*_x_ = 3.713 Mg m^−^^3^
Monoclinic, *C*2/*c*	Mo *K*α radiation, λ = 0.71073 Å
*a* = 11.9796 (13) Å	Cell parameters from 2076 reflections
*b* = 12.6935 (13) Å	θ = 2.5–35.6°
*c* = 6.5239 (7) Å	µ = 3.82 mm^−^^1^
β = 114.555 (3)°	*T* = 296 K
*V* = 902.33 (17) Å^3^	Block, colourless
*Z* = 4	0.31 × 0.24 × 0.20 mm

### Sodium magnesium indium(III) tris(orthophosphate) (I) Data collection

**Table:**

Bruker X8 APEXII diffractometer	2076 independent reflections
Radiation source: fine-focus sealed tube	2012 reflections with *I* > 2σ(*I*)
Graphite monochromator	*R*_int_ = 0.026
φ and ω scans	θ_max_ = 35.6°, θ_min_ = 2.5°
Absorption correction: multi-scan (SADABS; Krause *et al.*, 2015)	*h* = −19→19
*T*_min_ = 0.596, *T*_max_ = 0.748	*k* = −20→20
21364 measured reflections	*l* = −10→4

### Sodium magnesium indium(III) tris(orthophosphate) (I) Refinement

**Table:**

Refinement on *F*^2^	97 parameters
Least-squares matrix: full	0 restraints
*R*[*F*^2^ > 2σ(*F*^2^)] = 0.024	*w* = 1/[σ^2^(*F*_o_^2^) + (0.0082*P*)^2^ + 5.6344*P*] where *P* = (*F*_o_^2^ + 2*F*_c_^2^)/3
*wR*(*F*^2^) = 0.058	(Δ/σ)_max_ = 0.001
*S* = 1.29	Δρ_max_ = 0.64 e Å^−^^3^
2076 reflections	Δρ_min_ = −0.88 e Å^−^^3^

### Sodium magnesium indium(III) tris(orthophosphate) (I) Special details

**Table:**

Geometry. All esds (except the esd in the dihedral angle between two l.s. planes) are estimated using the full covariance matrix. The cell esds are taken into account individually in the estimation of esds in distances, angles and torsion angles; correlations between esds in cell parameters are only used when they are defined by crystal symmetry. An approximate (isotropic) treatment of cell esds is used for estimating esds involving l.s. planes.

### Sodium magnesium indium(III) tris(orthophosphate) (I) Fractional atomic coordinates and isotropic or equivalent isotropic displacement parameters (Å^2^)

**Table:**

	*x*	*y*	*z*	*U*_iso_*/*U*_eq_	Occ. (<1)
Mg1	0.71903 (3)	0.84384 (2)	0.13247 (5)	0.00578 (6)	0.68
In1	0.71903 (3)	0.84384 (2)	0.13247 (5)	0.00578 (6)	0.32
In2	0.500000	0.73266 (2)	0.250000	0.00619 (6)	0.51
Mg2	0.500000	0.73266 (2)	0.250000	0.00619 (6)	0.49
P1	0.76657 (4)	0.60997 (4)	0.37446 (8)	0.00665 (8)	
P2	0.500000	0.29168 (6)	0.250000	0.00702 (11)	
Na1	0.500000	0.500000	0.000000	0.0261 (4)	
Na2	1.000000	0.4813 (2)	0.750000	0.0369 (8)	0.848 (9)
O1	0.77790 (14)	0.67695 (12)	0.1877 (2)	0.0092 (2)	
O2	0.83997 (14)	0.66480 (12)	0.6061 (2)	0.0096 (2)	
O3	0.82556 (15)	0.50207 (12)	0.3858 (3)	0.0126 (3)	
O4	0.62982 (14)	0.60255 (13)	0.3291 (3)	0.0128 (3)	
O5	0.59943 (14)	0.36515 (12)	0.2450 (3)	0.0124 (3)	
O6	0.45840 (13)	0.22035 (12)	0.0372 (2)	0.0099 (2)	

### Sodium magnesium indium(III) tris(orthophosphate) (I) Atomic displacement parameters (Å^2^)

**Table:**

	*U*^11^	*U*^22^	*U*^33^	*U*^12^	*U*^13^	*U*^23^
Mg1	0.00607 (11)	0.00594 (11)	0.00620 (11)	−0.00056 (8)	0.00342 (8)	−0.00092 (8)
In1	0.00607 (11)	0.00594 (11)	0.00620 (11)	−0.00056 (8)	0.00342 (8)	−0.00092 (8)
In2	0.00700 (12)	0.00580 (11)	0.00657 (11)	0.000	0.00360 (9)	0.000
Mg2	0.00700 (12)	0.00580 (11)	0.00657 (11)	0.000	0.00360 (9)	0.000
P1	0.00876 (19)	0.00636 (18)	0.00521 (17)	−0.00120 (14)	0.00328 (15)	−0.00039 (14)
P2	0.0072 (3)	0.0077 (3)	0.0054 (2)	0.000	0.0018 (2)	0.000
Na1	0.0375 (9)	0.0121 (6)	0.0156 (6)	0.0017 (6)	−0.0020 (6)	0.0029 (5)
Na2	0.0228 (11)	0.0569 (17)	0.0235 (11)	0.000	0.0020 (8)	0.000
O1	0.0121 (6)	0.0098 (6)	0.0062 (5)	−0.0004 (5)	0.0044 (5)	0.0014 (4)
O2	0.0136 (6)	0.0099 (6)	0.0053 (5)	−0.0027 (5)	0.0039 (5)	−0.0017 (4)
O3	0.0189 (7)	0.0062 (5)	0.0135 (6)	0.0003 (5)	0.0077 (5)	−0.0009 (5)
O4	0.0109 (6)	0.0136 (6)	0.0156 (7)	−0.0035 (5)	0.0072 (5)	−0.0016 (5)
O5	0.0090 (6)	0.0111 (6)	0.0155 (7)	−0.0012 (5)	0.0036 (5)	0.0053 (5)
O6	0.0083 (5)	0.0131 (6)	0.0076 (5)	0.0007 (5)	0.0026 (4)	−0.0026 (5)

### Sodium magnesium indium(III) tris(orthophosphate) (I) Geometric parameters (Å, º)

**Table:**

Mg1—O5^i^	1.9992 (16)	P2—O6^v^	1.5558 (15)
Mg1—O3^i^	2.0690 (16)	P2—O6	1.5558 (15)
Mg1—O2^ii^	2.1030 (15)	Na1—O5^iii^	2.3068 (15)
Mg1—O6^iii^	2.1099 (15)	Na1—O5	2.3068 (15)
Mg1—O1^iv^	2.1207 (14)	Na1—O4	2.4387 (16)
Mg1—O1	2.2144 (15)	Na1—O4^iii^	2.4388 (16)
In2—O4^v^	2.1783 (17)	Na1—O4^viii^	2.6072 (15)
In2—O4	2.1784 (17)	Na1—O4^v^	2.6072 (15)
In2—O2^ii^	2.1807 (15)	Na1—O5^viii^	2.9603 (17)
In2—O2^vi^	2.1807 (15)	Na1—O5^v^	2.9603 (17)
In2—O6^vii^	2.2115 (15)	Na2—O3	2.4401 (17)
In2—O6^iii^	2.2115 (15)	Na2—O3^ix^	2.4401 (17)
P1—O3	1.5287 (16)	Na2—O3^x^	2.5955 (17)
P1—O1	1.5373 (15)	Na2—O3^vii^	2.5955 (17)
P1—O4	1.5419 (16)	Na2—O6^xi^	2.856 (3)
P1—O2	1.5609 (15)	Na2—O6^xii^	2.856 (3)
P2—O5	1.5238 (16)	Na2—O2	2.913 (3)
P2—O5^v^	1.5238 (16)	Na2—O2^ix^	2.913 (3)
			
O5^i^—Mg1—O3^i^	95.94 (6)	O5—Na1—O4^viii^	72.41 (6)
O5^i^—Mg1—O2^ii^	111.05 (6)	O4—Na1—O4^viii^	111.56 (7)
O3^i^—Mg1—O2^ii^	86.09 (6)	O4^iii^—Na1—O4^viii^	68.44 (7)
O5^i^—Mg1—O6^iii^	162.43 (6)	O5^iii^—Na1—O4^v^	72.41 (6)
O3^i^—Mg1—O6^iii^	99.50 (6)	O5—Na1—O4^v^	107.59 (6)
O2^ii^—Mg1—O6^iii^	78.50 (6)	O4—Na1—O4^v^	68.44 (7)
O5^i^—Mg1—O1^iv^	87.10 (6)	O4^iii^—Na1—O4^v^	111.56 (7)
O3^i^—Mg1—O1^iv^	100.00 (6)	O4^viii^—Na1—O4^v^	180.0
O2^ii^—Mg1—O1^iv^	160.31 (6)	O5^iii^—Na1—O5^viii^	52.70 (6)
O6^iii^—Mg1—O1^iv^	82.03 (6)	O5—Na1—O5^viii^	127.30 (6)
O5^i^—Mg1—O1	81.05 (6)	O4—Na1—O5^viii^	85.86 (5)
O3^i^—Mg1—O1	174.39 (6)	O4^iii^—Na1—O5^viii^	94.14 (5)
O2^ii^—Mg1—O1	90.57 (6)	O4^viii^—Na1—O5^viii^	66.27 (5)
O6^iii^—Mg1—O1	84.22 (6)	O4^v^—Na1—O5^viii^	113.73 (5)
O1^iv^—Mg1—O1	84.63 (6)	O5^iii^—Na1—O5^v^	127.30 (6)
O4^v^—In2—O4	81.40 (8)	O5—Na1—O5^v^	52.70 (6)
O4^v^—In2—O2^ii^	165.44 (6)	O4—Na1—O5^v^	94.14 (5)
O4—In2—O2^ii^	86.40 (6)	O4^iii^—Na1—O5^v^	85.86 (5)
O4^v^—In2—O2^vi^	86.40 (6)	O4^viii^—Na1—O5^v^	113.73 (5)
O4—In2—O2^vi^	165.44 (6)	O4^v^—Na1—O5^v^	66.27 (5)
O2^ii^—In2—O2^vi^	106.70 (8)	O5^viii^—Na1—O5^v^	180.0
O4^v^—In2—O6^vii^	90.87 (6)	O3—Na2—O3^ix^	167.61 (15)
O4—In2—O6^vii^	113.19 (6)	O3—Na2—O3^x^	98.29 (5)
O2^ii^—In2—O6^vii^	86.65 (5)	O3^ix^—Na2—O3^x^	80.70 (5)
O2^vi^—In2—O6^vii^	74.72 (5)	O3—Na2—O3^vii^	80.70 (5)
O4^v^—In2—O6^iii^	113.19 (6)	O3^ix^—Na2—O3^vii^	98.29 (5)
O4—In2—O6^iii^	90.87 (6)	O3^x^—Na2—O3^vii^	170.69 (14)
O2^ii^—In2—O6^iii^	74.72 (5)	O3—Na2—O6^xi^	73.60 (6)
O2^vi^—In2—O6^iii^	86.65 (5)	O3^ix^—Na2—O6^xi^	118.42 (10)
O6^vii^—In2—O6^iii^	148.70 (8)	O3^x^—Na2—O6^xi^	84.80 (7)
O3—P1—O1	110.04 (9)	O3^vii^—Na2—O6^xi^	103.66 (8)
O3—P1—O4	112.79 (9)	O3—Na2—O6^xii^	118.42 (10)
O1—P1—O4	108.57 (9)	O3^ix^—Na2—O6^xii^	73.60 (6)
O3—P1—O2	106.82 (9)	O3^x^—Na2—O6^xii^	103.66 (8)
O1—P1—O2	108.72 (8)	O3^vii^—Na2—O6^xii^	84.80 (7)
O4—P1—O2	109.83 (9)	O6^xi^—Na2—O6^xii^	52.60 (8)
O5—P2—O5^v^	104.53 (13)	O3—Na2—O2	54.35 (6)
O5—P2—O6^v^	114.33 (8)	O3^ix^—Na2—O2	114.22 (10)
O5^v^—P2—O6^v^	107.48 (9)	O3^x^—Na2—O2	109.91 (9)
O5—P2—O6	107.48 (9)	O3^vii^—Na2—O2	61.95 (6)
O5^v^—P2—O6	114.33 (8)	O6^xi^—Na2—O2	126.98 (4)
O6^v^—P2—O6	108.82 (12)	O6^xii^—Na2—O2	146.30 (5)
O5^iii^—Na1—O5	180.0	O3—Na2—O2^ix^	114.22 (10)
O5^iii^—Na1—O4	99.83 (6)	O3^ix^—Na2—O2^ix^	54.35 (6)
O5—Na1—O4	80.17 (6)	O3^x^—Na2—O2^ix^	61.95 (6)
O5^iii^—Na1—O4^iii^	80.17 (6)	O3^vii^—Na2—O2^ix^	109.91 (9)
O5—Na1—O4^iii^	99.83 (6)	O6^xi^—Na2—O2^ix^	146.30 (5)
O4—Na1—O4^iii^	180.0	O6^xii^—Na2—O2^ix^	126.98 (4)
O5^iii^—Na1—O4^viii^	107.59 (6)	O2—Na2—O2^ix^	73.83 (9)

Symmetry codes: (i) −*x*+3/2, *y*+1/2, −*z*+1/2; (ii) −*x*+3/2, −*y*+3/2, −*z*+1; (iii) −*x*+1, −*y*+1, −*z*; (iv) −*x*+3/2, −*y*+3/2, −*z*; (v) −*x*+1, *y*, −*z*+1/2; (vi) *x*−1/2, −*y*+3/2, *z*−1/2; (vii) *x*, −*y*+1, *z*+1/2; (viii) *x*, −*y*+1, *z*−1/2; (ix) −*x*+2, *y*, −*z*+3/2; (x) −*x*+2, −*y*+1, −*z*+1; (xi) *x*+1/2, −*y*+1/2, *z*+1/2; (xii) −*x*+3/2, −*y*+1/2, −*z*+1.

### Silver magnesium indium(III) tris(orthophosphate) (II) Crystal data

**Table:**

Ag_1.69_Mg_1.69_In_1.31_(PO_4_)_3_	*F*(000) = 1219
*M**_r_* = 658.40	*D*_x_ = 4.794 Mg m^−^^3^
Monoclinic, *C*2/*c*	Mo *K*α radiation, λ = 0.71073 Å
*a* = 12.0273 (3) Å	Cell parameters from 1827 reflections
*b* = 12.8120 (3) Å	θ = 2.5–33.7°
*c* = 6.5061 (2) Å	µ = 7.59 mm^−^^1^
β = 114.519 (1)°	*T* = 296 K
*V* = 912.14 (4) Å^3^	Block, colourless
*Z* = 4	0.30 × 0.27 × 0.23 mm

### Silver magnesium indium(III) tris(orthophosphate) (II) Data collection

**Table:**

Bruker X8 APEXII diffractometer	1827 independent reflections
Radiation source: fine-focus sealed tube	1818 reflections with *I* > 2σ(*I*)
Graphite monochromator	*R*_int_ = 0.027
φ and ω scans	θ_max_ = 33.7°, θ_min_ = 2.5°
Absorption correction: multi-scan (SADABS; Krause *et al.*, 2015)	*h* = −18→18
*T*_min_ = 0.404, *T*_max_ = 0.748	*k* = −20→20
13615 measured reflections	*l* = −10→7

### Silver magnesium indium(III) tris(orthophosphate) (II) Refinement

**Table:**

Refinement on *F*^2^	0 restraints
Least-squares matrix: full	*w* = 1/[σ^2^(*F*_o_^2^) + (0.0071*P*)^2^ + 8.2996*P*] where *P* = (*F*_o_^2^ + 2*F*_c_^2^)/3
*R*[*F*^2^ > 2σ(*F*^2^)] = 0.022	(Δ/σ)_max_ = 0.001
*wR*(*F*^2^) = 0.053	Δρ_max_ = 2.32 e Å^−^^3^
*S* = 1.25	Δρ_min_ = −1.36 e Å^−^^3^
1827 reflections	Extinction correction: SHELXL2016 (Sheldrick, 2015*b*), Fc^*^=kFc[1+0.001xFc^2^λ^3^/sin(2θ)]^-1/4^
97 parameters	Extinction coefficient: 0.00143 (15)

### Silver magnesium indium(III) tris(orthophosphate) (II) Special details

**Table:**

Geometry. All esds (except the esd in the dihedral angle between two l.s. planes) are estimated using the full covariance matrix. The cell esds are taken into account individually in the estimation of esds in distances, angles and torsion angles; correlations between esds in cell parameters are only used when they are defined by crystal symmetry. An approximate (isotropic) treatment of cell esds is used for estimating esds involving l.s. planes.

### Silver magnesium indium(III) tris(orthophosphate) (II) Fractional atomic coordinates and isotropic or equivalent isotropic displacement parameters (Å^2^)

**Table:**

	*x*	*y*	*z*	*U*_iso_*/*U*_eq_	Occ. (<1)
In1	0.71633 (3)	0.84600 (3)	0.12503 (6)	0.0061 (2)	0.314 (2)
Mg1	0.71633 (3)	0.84600 (3)	0.12503 (6)	0.0061 (2)	0.687 (2)
Mg2	0.500000	0.73554 (2)	0.250000	0.00583 (9)	0.314 (2)
In2	0.500000	0.73554 (2)	0.250000	0.00583 (9)	0.686 (2)
Ag1	0.500000	0.500000	0.000000	0.02109 (10)	
Ag2	1.000000	0.48627 (5)	0.750000	0.03258 (15)	0.6988
P1	0.76583 (5)	0.61258 (4)	0.37509 (9)	0.00362 (10)	
P2	0.500000	0.29241 (6)	0.250000	0.00404 (13)	
O1	0.77898 (15)	0.67881 (13)	0.1901 (3)	0.0064 (3)	
O2	0.84069 (15)	0.66448 (13)	0.6096 (3)	0.0065 (3)	
O3	0.81775 (16)	0.50301 (13)	0.3825 (3)	0.0096 (3)	
O4	0.62999 (15)	0.60993 (13)	0.3340 (3)	0.0080 (3)	
O5	0.60107 (15)	0.36446 (13)	0.2501 (3)	0.0094 (3)	
O6	0.45894 (15)	0.22207 (13)	0.0360 (3)	0.0062 (3)	

### Silver magnesium indium(III) tris(orthophosphate) (II) Atomic displacement parameters (Å^2^)

**Table:**

	*U*^11^	*U*^22^	*U*^33^	*U*^12^	*U*^13^	*U*^23^
In1	0.00587 (16)	0.00690 (16)	0.00638 (17)	−0.00070 (10)	0.00336 (11)	−0.00109 (10)
Mg1	0.00587 (16)	0.00690 (16)	0.00638 (17)	−0.00070 (10)	0.00336 (11)	−0.00109 (10)
Mg2	0.00560 (13)	0.00642 (13)	0.00604 (13)	0.000	0.00297 (9)	0.000
In2	0.00560 (13)	0.00642 (13)	0.00604 (13)	0.000	0.00297 (9)	0.000
Ag1	0.03303 (18)	0.00978 (13)	0.01314 (14)	0.00397 (10)	0.00229 (11)	0.00201 (9)
Ag2	0.0127 (2)	0.0291 (3)	0.0406 (3)	0.000	−0.00429 (19)	0.000
P1	0.0037 (2)	0.0038 (2)	0.0036 (2)	−0.00040 (15)	0.00170 (17)	−0.00038 (15)
P2	0.0035 (3)	0.0051 (3)	0.0031 (3)	0.000	0.0010 (2)	0.000
O1	0.0069 (6)	0.0084 (6)	0.0043 (6)	−0.0004 (5)	0.0027 (5)	0.0011 (5)
O2	0.0084 (6)	0.0072 (6)	0.0034 (6)	−0.0025 (5)	0.0020 (5)	−0.0018 (5)
O3	0.0106 (7)	0.0043 (6)	0.0142 (8)	0.0006 (5)	0.0055 (6)	−0.0022 (5)
O4	0.0052 (6)	0.0078 (6)	0.0120 (7)	0.0002 (5)	0.0045 (6)	0.0009 (5)
O5	0.0055 (6)	0.0091 (7)	0.0129 (8)	−0.0018 (5)	0.0032 (6)	0.0031 (6)
O6	0.0052 (6)	0.0091 (6)	0.0043 (6)	−0.0006 (5)	0.0019 (5)	−0.0014 (5)

### Silver magnesium indium(III) tris(orthophosphate) (II) Geometric parameters (Å, º)

**Table:**

In1—O5^i^	2.0146 (17)	Ag1—O5^viii^	2.9625 (19)
In1—O3^i^	2.0495 (17)	Ag1—O5^v^	2.9625 (19)
In1—O1^ii^	2.0985 (16)	Ag2—O3	2.4934 (19)
In1—O2^iii^	2.1098 (17)	Ag2—O3^ix^	2.4934 (19)
In1—O6^iv^	2.1140 (16)	Ag2—O3^x^	2.6713 (18)
In1—O1	2.2518 (17)	Ag2—O3^vii^	2.6713 (18)
Mg2—O4	2.1502 (17)	Ag2—O2^ix^	2.8751 (18)
Mg2—O4^v^	2.1503 (17)	Ag2—O2	2.8751 (18)
Mg2—O2^iii^	2.1665 (16)	Ag2—O6^xi^	2.9558 (18)
Mg2—O2^vi^	2.1665 (16)	Ag2—O6^xii^	2.9558 (18)
Mg2—O6^vii^	2.1827 (16)	P1—O3	1.5290 (17)
Mg2—O6^iv^	2.1827 (16)	P1—O1	1.5335 (17)
Ag1—O5	2.3450 (17)	P1—O4	1.5425 (17)
Ag1—O5^iv^	2.3451 (17)	P1—O2	1.5624 (17)
Ag1—O4^iv^	2.5162 (17)	P2—O5^v^	1.5261 (17)
Ag1—O4	2.5162 (17)	P2—O5	1.5261 (17)
Ag1—O4^viii^	2.6449 (17)	P2—O6	1.5569 (17)
Ag1—O4^v^	2.6449 (17)	P2—O6^v^	1.5569 (17)
			
O5^i^—In1—O3^i^	93.91 (7)	O4^viii^—Ag1—O5^viii^	68.99 (5)
O5^i^—In1—O1^ii^	86.84 (7)	O4^v^—Ag1—O5^viii^	111.01 (5)
O3^i^—In1—O1^ii^	102.20 (7)	O5—Ag1—O5^v^	52.97 (7)
O5^i^—In1—O2^iii^	110.35 (7)	O5^iv^—Ag1—O5^v^	127.03 (7)
O3^i^—In1—O2^iii^	87.27 (7)	O4^iv^—Ag1—O5^v^	84.06 (5)
O1^ii^—In1—O2^iii^	159.96 (6)	O4—Ag1—O5^v^	95.94 (5)
O5^i^—In1—O6^iv^	160.79 (7)	O4^viii^—Ag1—O5^v^	111.01 (5)
O3^i^—In1—O6^iv^	104.16 (7)	O4^v^—Ag1—O5^v^	68.99 (5)
O1^ii^—In1—O6^iv^	83.05 (6)	O5^viii^—Ag1—O5^v^	180.0
O2^iii^—In1—O6^iv^	77.49 (6)	O3—Ag2—O3^ix^	170.14 (8)
O5^i^—In1—O1	79.17 (7)	O3—Ag2—O3^x^	101.46 (5)
O3^i^—In1—O1	170.47 (7)	O3^ix^—Ag2—O3^x^	78.02 (5)
O1^ii^—In1—O1	84.09 (6)	O3—Ag2—O3^vii^	78.02 (5)
O2^iii^—In1—O1	89.01 (6)	O3^ix^—Ag2—O3^vii^	101.46 (5)
O6^iv^—In1—O1	83.56 (6)	O3^x^—Ag2—O3^vii^	174.11 (7)
O4—Mg2—O4^v^	83.10 (9)	O3—Ag2—O2^ix^	116.17 (5)
O4—Mg2—O2^iii^	85.00 (6)	O3^ix^—Ag2—O2^ix^	54.71 (5)
O4^v^—Mg2—O2^iii^	166.46 (6)	O3^x^—Ag2—O2^ix^	62.21 (5)
O4—Mg2—O2^vi^	166.46 (6)	O3^vii^—Ag2—O2^ix^	112.62 (5)
O4^v^—Mg2—O2^vi^	84.99 (6)	O3—Ag2—O2	54.71 (5)
O2^iii^—Mg2—O2^vi^	107.51 (9)	O3^ix^—Ag2—O2	116.17 (5)
O4—Mg2—O6^vii^	111.55 (6)	O3^x^—Ag2—O2	112.62 (5)
O4^v^—Mg2—O6^vii^	90.29 (6)	O3^vii^—Ag2—O2	62.21 (5)
O2^iii^—Mg2—O6^vii^	88.11 (6)	O2^ix^—Ag2—O2	74.85 (7)
O2^vi^—Mg2—O6^vii^	74.86 (6)	O3—Ag2—O6^xi^	73.59 (5)
O4—Mg2—O6^iv^	90.29 (6)	O3^ix^—Ag2—O6^xi^	115.97 (5)
O4^v^—Mg2—O6^iv^	111.55 (6)	O3^x^—Ag2—O6^xi^	83.88 (5)
O2^iii^—Mg2—O6^iv^	74.86 (6)	O3^vii^—Ag2—O6^xi^	101.50 (5)
O2^vi^—Mg2—O6^iv^	88.11 (6)	O2^ix^—Ag2—O6^xi^	145.67 (5)
O6^vii^—Mg2—O6^iv^	151.18 (9)	O2—Ag2—O6^xi^	127.48 (4)
O4—Mg2—O5^iv^	83.64 (6)	O3—Ag2—O6^xii^	115.97 (5)
O5—Ag1—O5^iv^	180.0	O3^ix^—Ag2—O6^xii^	73.59 (5)
O5—Ag1—O4^iv^	98.18 (6)	O3^x^—Ag2—O6^xii^	101.50 (5)
O5^iv^—Ag1—O4^iv^	81.82 (6)	O3^vii^—Ag2—O6^xii^	83.88 (5)
O5—Ag1—O4	81.82 (6)	O2^ix^—Ag2—O6^xii^	127.48 (4)
O5^iv^—Ag1—O4	98.17 (6)	O2—Ag2—O6^xii^	145.67 (5)
O4^iv^—Ag1—O4	180.0	O6^xi^—Ag2—O6^xii^	50.87 (6)
O5—Ag1—O4^viii^	70.42 (6)	O3—P1—O1	111.04 (10)
O5^iv^—Ag1—O4^viii^	109.58 (6)	O3—P1—O4	112.00 (10)
O4^iv^—Ag1—O4^viii^	67.05 (7)	O1—P1—O4	108.85 (9)
O4—Ag1—O4^viii^	112.95 (7)	O3—P1—O2	107.32 (10)
O5—Ag1—O4^v^	109.58 (6)	O1—P1—O2	109.01 (9)
O5^iv^—Ag1—O4^v^	70.42 (6)	O4—P1—O2	108.55 (10)
O4^iv^—Ag1—O4^v^	112.95 (7)	O5^v^—P2—O5	105.56 (14)
O4—Ag1—O4^v^	67.05 (7)	O5^v^—P2—O6	113.12 (9)
O4^viii^—Ag1—O4^v^	180.00 (5)	O5—P2—O6	107.91 (9)
O5—Ag1—O5^viii^	127.03 (7)	O5^v^—P2—O6^v^	107.91 (9)
O5^iv^—Ag1—O5^viii^	52.97 (7)	O5—P2—O6^v^	113.12 (9)
O4^iv^—Ag1—O5^viii^	95.94 (5)	O6—P2—O6^v^	109.26 (13)
O4—Ag1—O5^viii^	84.06 (5)		

Symmetry codes: (i) −*x*+3/2, *y*+1/2, −*z*+1/2; (ii) −*x*+3/2, −*y*+3/2, −*z*; (iii) −*x*+3/2, −*y*+3/2, −*z*+1; (iv) −*x*+1, −*y*+1, −*z*; (v) −*x*+1, *y*, −*z*+1/2; (vi) *x*−1/2, −*y*+3/2, *z*−1/2; (vii) *x*, −*y*+1, *z*+1/2; (viii) *x*, −*y*+1, *z*−1/2; (ix) −*x*+2, *y*, −*z*+3/2; (x) −*x*+2, −*y*+1, −*z*+1; (xi) *x*+1/2, −*y*+1/2, *z*+1/2; (xii) −*x*+3/2, −*y*+1/2, −*z*+1.
